# First-in-human study to investigate the safety and pharmacokinetics of salvianolic acid A and pharmacokinetic simulation using a physiologically based pharmacokinetic model

**DOI:** 10.3389/fphar.2022.907208

**Published:** 2022-11-04

**Authors:** Jinliang Chen, Zourong Ruan, Honggang Lou, Dandan Yang, Rong Shao, Yichao Xu, Xinhua Hu, Bo Jiang

**Affiliations:** Center of Clinical Pharmacology, School of Medicine, The Second Affiliated Hospital of Zhejiang University, Hangzhou, Zhejiang, China

**Keywords:** salvianolic acid A, pharmacokinetics, safety, membrane transporter, physiologically based pharmacokinetic model

## Abstract

Salvianolic acid A (SAA) is a water-soluble phenolic acid component from *Salvia miltiorrhiza* Bunge currently under development for myocardial protection treatment for coronary heart disease (CHD). We investigated the safety, tolerability, and pharmacokinetics of single and multiple ascending doses of SAA. Additionally, a physiologically based pharmacokinetic (PBPK) model was developed to simulate the pharmacokinetics of SAA. This was a first-in-human (FIH), randomized, double-blind, placebo-controlled, single, and multiple-dose study in 116 healthy Chinese subjects with the range of 10–300 mg and 60–200 mg SAA, respectively. SAA was well tolerated at all dose levels, following both single and multiple doses, with a low overall incidence of treatment-emergent adverse events (TEAEs) which appeared to be no dose-related. The main pharmacokinetic parameter of SAA, assessed by the power model, was the lack of proportionality with the dose range after single dosing. The 90% CIs of the slope *β* of C_max_ (1.214 [1.150–1.278]) and AUC_0-t_ (1.222 [1.156–1.288]) were not within the predefined acceptance range, and the direction of the deviation was higher than expected. PBPK modeling suggested the transfer ability saturation of hepatic organic anion-transporting polypeptide 1B1 (OATP1B1) and P-glycoprotein (P-gp) might result in a relatively low distribution rate at higher doses. Clinical plasma concentrations observed were in good agreement with PBPK prediction. SAA showed well-characterized pharmacokinetics and was generally well tolerated in the dose range investigated. The PBPK model provides valuable pharmacokinetic knowledge for further clinical development.

## Introduction

Coronary heart disease (CHD) is the most common kind of cardiovascular disease resulting from decreased myocardial perfusion that causes angina due to ischemia and can result in myocardial infarction and/or heart failure (Olvera Lopez et al., 2021). Atherosclerosis can potentially cause CHD as a consequence of decreased or absent blood flow from stenosis of the blood vessels (Libby et al., 2011). In recent years, traditional Chinese medicine has been widely used in the treatment of CHD due to its fewer side effects. Salvianolic acid A (SAA) is a water-soluble phenolic acid monomer compound extracted from *Salvia miltiorrhiza* Bunge, a traditional Chinese medicine, known as Danshen (Li et al., 2018). SAA has the strongest pharmacological activity among the water-soluble components of Danshen (Wu et al., 2020). Various pharmacological effects, such as a strong antioxidant effect, protection of the heart against ischemia-reperfusion injury, significant protection of acute myocardial ischemia caused by drugs, inhibition of platelet aggregation, improvement in microcirculation, and the promotion of vasodilation, were observed (Lin et al., 1996; Tang et al., 2002; Pan et al., 2011).

The most amount of SAA exists in an ionic state in the intestinal tract, so it cannot easily pass through the intestinal biofilm, resulting in the relatively low bioavailability. Preclinical studies demonstrated that the oral absolute bioavailability of SAA in the rat is approximately 0.6%, and nearly no SAA in the plasma could be detected even after oral administration of 1000 mg in Beagle dogs (unpublished data). In another study, low oral bioavailability (1.25%) of SAA in Beagle dogs was found as well (Sun et al., 2013). Thus, SAA was developed as a sodium salt form for injection.

Physiologically based pharmacokinetic (PBPK) models represent the body as compartments parameterized based on the physiology of tissues and organs including composition, volumes, and blood flows (Miller et al., 2019), which can be used to integrate the numerous *in vitro* and *in silico* data generated for a compound and to add value by transforming these properties into the potential *in vivo* outcomes (Parrott et al., 2013). It has been already routinely used during drug discovery for *in vitro* to *in vivo* translation and pharmacokinetics modeling in preclinical species, leading to the application of verified models for first-in-human pharmacokinetic predictions (Gao et al., 2015; Kong et al., 2020). It is a powerful technique employed during early drug discovery.

In this first-in-human study, we aimed to investigate the safety, tolerability, and pharmacokinetics of single and multiple ascending doses of SAA in healthy subjects. The PBPK model was also included to study the potential distribution mechanism and predict the pharmacokinetic profile after intravenous infusion of SAA.

## Materials and methods

### Chemicals

Salvianolic acid A sodium salt (purity 95.2%) was provided by Chiatai Qingchunbao Pharmaceutical Co., Ltd. (Hangzhou, China). The isotope-labeled internal standard sodium salvianolic acid A-^18^O_2_ (purity 95%) was obtained from RISEN Pharmaceutical Co., Ltd. (Suzhou, China). Purified and deionized water was prepared using a Milli-Q water purification system (Millipore Corp. MA, United States). All the other reagents were of high-performance liquid chromatographic or analytic grade.

### Ethics

This study (www.chinadrugtrials.org.cn, CTR20181023) was performed at the Center of Clinical Pharmacology, the Second Affiliated Hospital of Zhejiang University School of Medicine, in compliance with the ethical principles founded in the Declaration of Helsinki and Good Clinical Practice Guidelines. The study protocol, amendments, informed consent forms, and the Investigator’s Brochure were reviewed and approved by the Human Subject Research Ethics Committee of the Second Affiliated Hospital of Zhejiang University School of Medicine. All subjects provided written informed consent.

### Study design

This is a first-in-human (FIH), randomized, double-blind, placebo-controlled, single, and multiple-dose study in healthy Chinese subjects. The study consisted of two parts: single ascending dose (Part A) and multiple ascending doses (Part B). In part A, subjects in each cohort (except cohort SG0 and SG1) were randomized according to the schedule prepared by SAS^®^ software (Version 9.1, SAS Institute, Inc. NC, United States), in a 5:1 ratio to receive SAA sodium salt for injection or placebo. Subjects in cohorts SG0 and SG1 received SAA sodium salt for injection only. Different doses of SAA sodium salt were prepared with 100 ml saline.

For part A, 92 subjects were enrolled in nine cohorts, and each subject received a single dose of SAA sodium salt for injection (10, 20, 40, 80, 120, 160, 200, 250, or 300 mg) or placebo by intravenous infusion for 60 min (except for cohort SG0 10 mg, the infusion time is 40 min). The starting dose (10 mg) was calculated using the no-observed-adverse-effect level (NOAEL) method in Beagle dogs, and the safety factor was set as 20. The higher-dose cohort would not be conducted unless the safety and tolerability of the previous dose level were reviewed and considered acceptable.

For part B, three cohorts of eight subjects were enrolled. Observing fast clearance of SAA in a single-dose study, twice daily (Q12H), doses were designed for multiple dosing. Each subject received Q12H doses of SAA sodium salt for injection (60, 120, or 200 mg) by intravenous infusion (60 min) for 5 days and once on day 6.

### Subjects

Chinese subjects (men and women) aged 18–45 years who have a body mass index (BMI) between 19.0 and 26.0 kg/m^2^ were eligible for the study. The subjects were considered healthy based on a detailed medical history, full physical examination, clinical laboratory test, 12-lead electrocardiogram (ECG), and vital signs. Subjects were required to be nonsmokers with no history of alcohol or drug abuse. Subjects had to have sufficient intelligence to understand the nature of the study and any hazards of participating in it and should have been willing to give written informed consent and adhere to study restrictions.

Subjects should not have been using any prescription medicine, over-the-counter medicine, vitamins, or herbal treatments during the 14 days before the screening. Subjects should also not have been using any alcohol-, caffeine-, or nicotine-containing products during the study period. For safety, subjects were excluded if the results of the SAA sodium skin test were positive.

### Safety and tolerability

The safety and tolerability assessments included treatment-emergent adverse events (TEAEs), physical examinations, vital signs, clinical laboratory tests, and 12-lead ECG. TEAEs were collected and recorded from the first dosing, continuing until the follow-up visit was finished. Vital signs and 12-lead ECG were recorded at regular intervals.

### Sample collection

For part A, blood samples (3.0 ml) for determination of plasma SAA concentrations were collected at pre-infusion, during the infusion period (15, 30, 45, and 60 min post-dose), and 5, 10, 30, and 45 min and 1.0, 1.5, 2.0, 3.0, 4.0, 6.0, 8.0, 10.0, and 12.0 h post-infusion.

Blood samples were collected during the infusion period (10, 20, 30, and 40 min post-dose) and additional 24.0 and 36.0 h post-infusion in the cohort SG0. In the cohort SG4, additional blood samples at 16.0 h post-infusion were collected.

For part B, blood samples were collected at pre-infusion, during the infusion period (15, 30, 45, and 60 min post-dose), and 5, 10, 30, and 45 min and 1.0, 1.5, 2.0, 3.0, 4.0, 6.0, 8.0, and 10.0 h post-infusion at first dosing on days 1 and 6. Blood samples were also collected prior to infusion and 3.0 h after infusion at each dosing on days 4 and 5. The schematic illustration of blood sampling is shown in Supplementary Figure S1.

Blood samples were taken into heparin sodium tubes and centrifuged (4°C, 3000 × g, 10 min) to separate the plasma. Plasma samples were mixed with equal volume vitamin C solution (200 μg/ml containing 1% formic acid) and then maintained at −70°C until analysis.

### Pharmacokinetic analysis

An ultraperformance liquid chromatography coupled with triple-quadrupole mass spectrometry (UPLC-MS/MS) method was developed and validated for the quantification of the SAA plasma concentration. Briefly, SAA was extracted from the plasma by protein precipitation using acetonitrile, and stable isotope-labeled sodium salvianolic acid A-^18^O_2_ was used as the internal standard (IS). The analytes were separated at a flow rate of 0.3 ml/min under gradient conditions using water containing 0.1% formic acid (mobile phase A) and acetonitrile containing 0.1% formic acid (mobile phase B) as the eluents. The gradient cycle consisted of an initial 1-min isocratic elution with 30% mobile phase B, followed by a linear increase in mobile phase B to 95% over another 1 min. The column was then eluted for 1 min with 95% mobile phase B before being eluted with 30% mobile phase B for the last 1 min. Quantification was performed using the multiple reaction monitoring modes to monitor the following transitions: *m/z* 493.1→295.1 for SAA and *m/z* 497.1→295.1 for IS. Four calibration ranges (2–2000 ng/ml, 4–6000 ng/ml, 10–6000 ng/ml, and 20–20000 ng/ml) were used for the quantification of plasma samples from various dose cohorts. The precision and accuracy of quality control (QC) samples met the following predefined acceptance criteria. The coefficient of variation (CV) of each QC level was within ±15% except for the lower limit of quantification (LLOQ), which was within ±20%. The accuracy of each QC level was within 85%–115% of the nominal concentration except for the LLOQ, which was within 80%–120%.

The following pharmacokinetic parameters were calculated using a non-compartmental method with WinNonlin™ software (Version 7.0, Pharsight Corp. CA, United States): the area under the plasma concentration–time curve (AUC) calculated from 0 to the last measurement point (AUC_0-t_), the AUC to infinity (AUC_0-inf_), and the AUC during a dosing interval (AUC_τ_); the maximum plasma concentration (C_max_) and the average plasma concentration during the dosing interval (C_avg_); terminal elimination half-life (t_1/2_); apparent clearance (CL); and the apparent volume of distribution (V_d_).

### Prediction of the exposure level of SAA in humans using the PBPK model

A PBPK model was developed to simulate the pharmacokinetics of SAA in humans using GastroPlus^®^ (Version 9.8, Simulations Plus, Inc., CA, United States). The physicochemical and ADME (absorption, distribution, metabolism, and excretion) properties of SAA were predicted by ADMET Predictor^®^ (Version 10.2, Simulations Plus, Inc., CA, United States ). The PBPK model was composed of 14 tissue compartments connected by venous and arterial blood. Hepatic organic anion-transporting polypeptide 1B1 (OATP1B1) and P-glycoprotein (P-gp) transporters were included in the PBPK model. All tissues were represented by a permeability-limited model. The key input data for the SAA PBPK model are listed in Supplementary Table S1. Tissue-to-plasma partition coefficients (K_p_ values) were estimated using a tissue composition-based equation taken from the Rodgers, Leahy, Rowland method. K_p_ values of the heart, liver, spleen, lung, kidney, and brain were calculated from the tissue distribution of SAA reported by Sun et al. (2018).

According to the proposal of the Extended Clearance Classification System (ECCS), SAA was classified to Class 3B-transporter-mediated hepatic uptake or renal clearance (low-permeability acid/zwitterions with MW > 400Da) (Varma et al., 2015). The assumptions for the SAA model were the following: SAA was predominantly eliminated *via* hepatobiliary excretion after intravenously dosing in humans; OATP1B1 contributed to the main hepatic uptake of SAA from blood in humans; P-gp was the dominant hepatic efflux transporters of SAA to bile; and the membrane permeation rate of SAA in other tissues was slow. The predicted human pharmacokinetics were compared with data obtained after single ascending doses, and the refined model was used to simulate the pharmacokinetic profile after multiple doses.

### Statistical analysis

To assess the dose proportionality of SAA, the pharmacokinetic parameters AUC_0-t_ and C_max_ were ln-transformed using the power model: ln(Y) = α+βln (dose), where Y is the pharmacokinetic parameter. The proportionality relationship between pharmacokinetic parameters and dose was considered if 90% confidence intervals (CIs) of the slope *β* were within the predefined range:

$$\left[1+\frac{\ln\left(\theta L\right)}{\ln\left(\theta R\right)},1+\frac{\ln\left(\theta H\right)}{\ln\left(\theta R\right)}\;\right],$$

where θL = 0.8, θH = 1.25, and R is the ratio of the highest dose to the lowest dose. Statistical analyses were performed by SPSS statistics software (Version 26, IBM Corp. NY, United States).

## Results

### Subjects

In part A, 98 subjects were enrolled in the study and 92 subjects received a single dose of SAA sodium salt for injection (*n* = 78) or placebo (*n* = 14). The mean (range) age was 27.9 years (18–43), body weight was 61.2 kg (47.2–83.3), and the body mass index (BMI) was 22.5 kg/m^2^ (19.2–26.0). Due to diarrhea or abnormal ECG results at baseline, six subjects were withdrawn and replaced before administration according to the protocol.

In part B, 27 subjects were enrolled, and finally, 24 subjects received multiple doses of SAA sodium salt for injection for 6 days. The mean (range) age was 30.0 years (22–41), body weight was 63.0 kg (47.5–78.2), and BMI was 22.8 kg/m^2^ (20.2–25.9). Due to abnormal ECG results or vital signs at baseline, three subjects were withdrawn and replaced before administration.

All subjects who received SAA completed the study, and the demographics of the subjects are presented in Table 1.

**TABLE 1:** Subject demographics.

Part A	Placebo	10 mg	20 mg	40 mg	80 mg	120 mg	160 mg	200 mg	250 mg	300 mg	Total
	*n* = 14	*n* = 2	*n* = 6	*n* = 10	*n* = 10	*n* = 10	*n* = 10	*n* = 10	*n* = 10	*n* = 10	*n* = 92
Age, years	29.5 (6.2)	25.5 (6.4)	27.2 (8.4)	29.0 (5.4)	20.0 (5.2)	29.9 (6.5)	28.0 (7.7)	23.6 (5.1)	27.9 (3.8)	28.7 (6.8)	27.9 (6.1)
Male, *n* (%)	5 (35.7)	1 (50.0)	3 (50.0)	5 (50.0)	5 (50.0)	5 (50.0)	5 (50.0)	6 (60.0)	4 (40.0)	5 (50.0)	46 (50.0)
BMI, kg/m^2^	22.8 (2.1)	22.5 (2.5)	23.7 (2.7)	22.7 (2.3)	20.8 (1.3)	23.1 (1.8)	22.8 (1.9)	22.2 (1.9)	22.4 (1.5)	22.7 (1.6)	22.5 (2.0)
Part B			60 mg Q12H			120 mg Q12H			200 mg Q12H		Total
			*n* = 8			*n* = 8			*n* = 8		*n* = 24
Age, years			29.1 (4.9)			35.5 (4.0)			25.5 (3.4)		30.0 (5.8)
Male, *n* (%)			4 (50.0)			5 (62.5)			4 (50.0)		13 (54.2)
BMI, kg/m^2^			23.2 (1.3)			22.7 (1.8)			22.4 (1.9)		22.8 (1.6)

Age and BMI are shown as mean (standard deviation).

BMI, body mass index; Q12H, dosing every 12 h.

### Clinical pharmacokinetics

**FIGURE 1:**
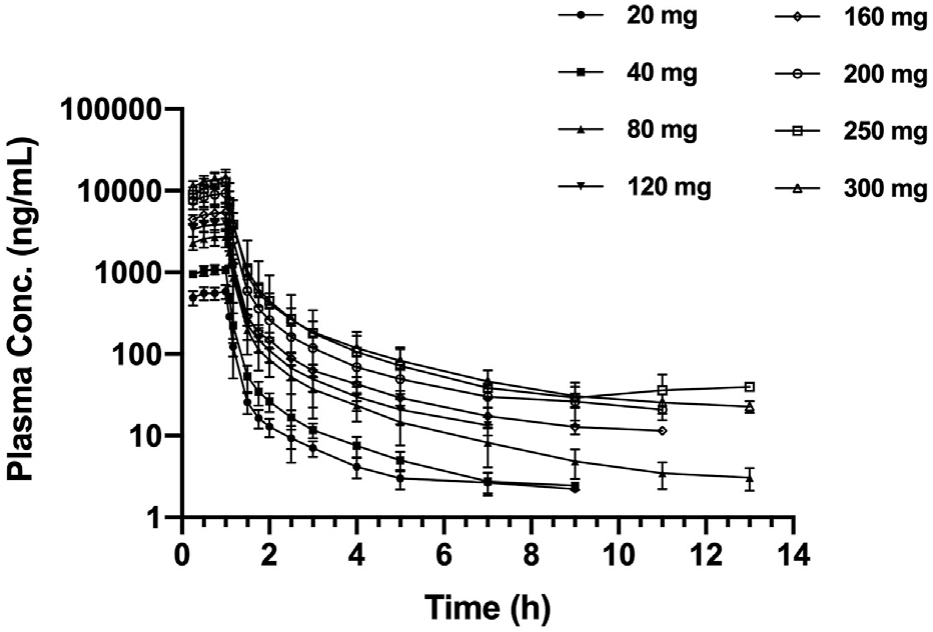
Mean (±SD) plasma concentration–time profiles, following the single ascending dose of SAA in healthy Chinese subjects. SAA, salvianolic acid A.

The mean SAA plasma concentration *versus* time curves in each cohort for a single dose are shown in Figure 1. Following a single dose, the plasma concentration increased immediately and reached the peak near or at the end of infusion. The plasma concentrations appeared to present a biphasic elimination profile with an initial rapid decline, followed by a gradual down terminal phase. The pharmacokinetic parameters are shown in Table 2. The mean terminal t_1/2_ ranged from 1.62 to 2.92 h, and the mean V_d_ ranged from 69.0 to 161.9 L. For each cohort, the pharmacokinetic profiles of male and female subjects are shown in Supplementary Figure S2. No sex difference was found after a single dose of SAA from 20 to 300 mg.

**TABLE 2:** Pharmacokinetic parameters in the single ascending dose study.

Cohort	Dose	C_max_	AUC_0-t_	AUC_0-inf_	t_1/2_	V_d_	CL
	(mg)	(ng/ml)	(h*ng/ml)	(h*ng/ml)	(h)	(L)	(L/h)
SG 0	10 (*n* = 2)	643 (266)	143 (114)	148 (118)	1.67 (1.33)	161.9 (0.9)	98.4 (78.1)
SG 1	20 (*n* = 6)	599 (100)	580 (103)	586 (105)	1.73 (0.86)	82.5 (29.2)	35.0 (6.2)
SG 2	40 (*n* = 10)	1124 (122)	1106 (126)	1114 (126)	2.20 (0.77)	115.8 (46.6)	36.3 (3.5)
SG 3	80 (*n* = 10)	2770 (656)	2920 (716)	2932 (719)	2.83 (0.43)	116.2 (27.6)	28.7 (6.5)
SG 4	120 (*n* = 10)	3925 (679)	4066 (788)	4101 (793)	1.62 (0.41)	69.0 (16.5)	30.2 (5.6)
SG 5	160 (*n* = 10)	5492 (966)	5505 (940)	5550 (942)	2.55 (0.52)	108.1 (25.5)	29.5 (4.3)
SG 6	200 (*n* = 10)	9344 (2382)	9450 (2580)	9530 (2590)	2.26 (0.43)	70.9 (15.6)	22.4 (6.1)
SG 7	250 (*n* = 10)	12,730 (5548)	12,620 (6030)	12,710 (6070)	2.39 (0.55)	73.4 (20.4)	22.0 (5.7)
SG 8	300 (*n* = 10)	14,310 (2738)	14,560 (2970)	14,660 (2980)	2.92 (0.50)	87.7 (14.1)	21.2 (4.1)

C_max_, maximum plasma concentration; AUC_0-t_, area under the plasma concentration–time curve from 0 to the last measurement time; AUC_0-inf_, area under the plasma concentration–time curve from 0 to infinity; t_1/2_, terminal elimination half-life; V_d_, apparent volume of distribution; CL, apparent clearance.

The mean SAA plasma concentration *versus* time curves in each cohort for multiple doses are shown in Supplementary Figure S3. The differences among the trough concentrations or the concentrations 3 h post-dosing, on days 4–6, were small in all three dose cohorts, indicating steady-state conditions appeared to be attained at least by day 4. The SAA exposures on day 6 after multiple administrations were similar to those of the first dosing on day 1. The multiple doses of pharmacokinetic parameters are shown in Table 3. The mean accumulation index ranged from 1.08 to 1.17, indicating little accumulation was observed after administrating SAA twice daily for 5 days.

**TABLE 3:** Pharmacokinetic parameters in the multiple ascending dose study.

Parameter	Cohort RG 1 60 mg Q12H (*n* = 8)		Cohort RG 2 120 mg Q12H (*n* = 8)		Cohort RG 3 200 mg Q12H (*n* = 8)	
	Day 1	Day 6	Day 1	Day 6	Day 1	Day 6
C_max_ (ng/ml)	2063 (264)	2149 (325)	3513 (494)	3561 (463)	7109 (1755)	6806 (1637)
AUC_0-t_ (h*ng/ml)	2098 (265)	2126 (347)	3545 (490)	3601 (557)	7137 (1999)	6954 (1832)
AUC_0-inf_ (h*ng/ml)	-	2164 (370)	-	3647 (571)	-	7048 (1859)
t_1/2_ (h)	2.34 (1.21)	3.40 (1.11)	3.11 (0.70)	4.29 (0.89)	1.57 (0.69)	2.87 (1.26)
C_avg_ (ng/ml)	-	178 (29)	-	301 (47)	-	583 (152)
AUC_τ_ (h*ng/ml)	-	2136 (347)	-	3607 (558)	-	6999 (1819)
Fluctuation (%)	-	1208 (72)	-	1187 (49)	-	1167 (36)
Accumulation index	-	1.11 (0.08)	-	1.17 (0.07)	-	1.08 (0.07)

Q12H, dosing every 12 h; C_max_, maximum plasma concentration; C_avg_, average plasma concentration during the dosing interval; AUC_0-t_, area under the plasma concentration–time curve from 0 to the last measurement time; AUC_0-inf_, area under the plasma concentration–time curve from 0 to infinity; AUC_τ_, area under the plasma concentration–time curve from the time of dosing to the start of the next dosing interval; t_1/2_, terminal elimination half-life.

Fluctuation (%) = $\frac{C_{\max}-C_{\min}}{C_{avg}}\times 100$ (C_min_, minimum plasma concentration between the dose time and dose time + τ).

Accumulation Index = $\frac{1}{1-e^{-\lambda_{z}\tau }}$ (*λ*_z_, terminal elimination rate constant).

**FIGURE 2:**
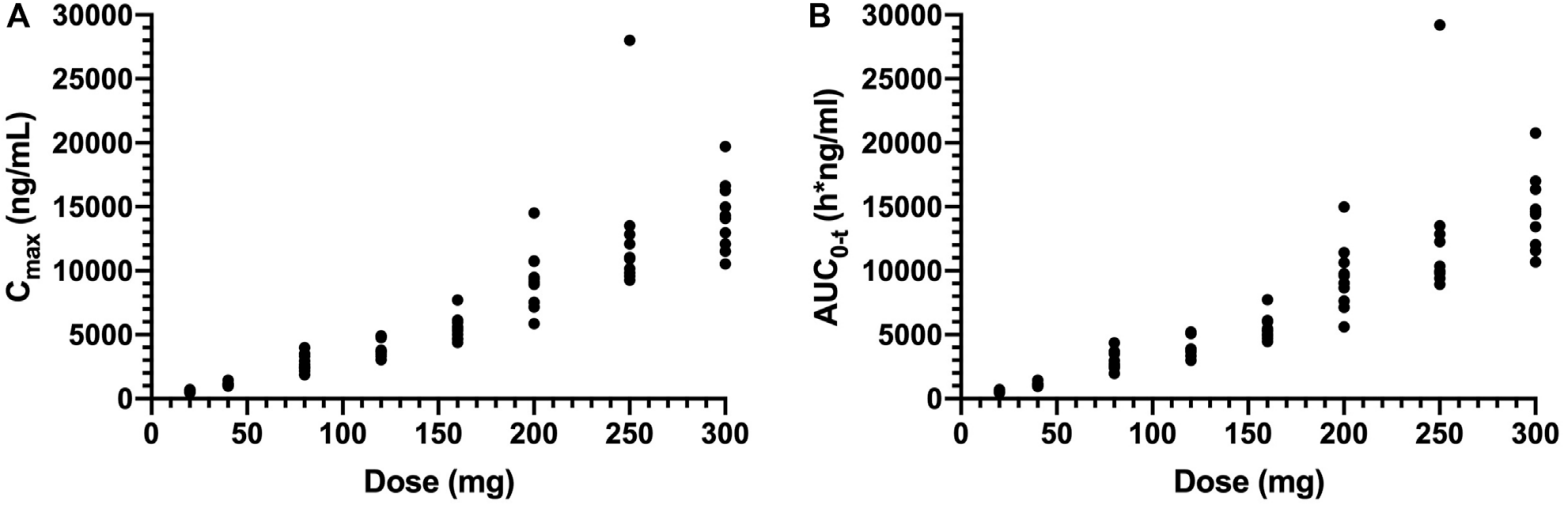
Plot of individual of C_max_ **(A)** and AUC_0-t_ **(B)** values *versus* dose in single ascending-dose study (20, 40, 80, 120, 160, 200, 250, and 300 mg). C_max_, the maximum plasma concentration; AUC_0-t_, the area under the plasma concentration–time curve from 0 to the last measurement time.

C_max_ and AUC_0-t_ values increased about 23.9-fold (means of 599–14310 ng/ml) and 25.1-fold (means of 580–14560 h*ng/ml) with a 15-fold (20–300 mg) increase in doses, respectively (Figure 2). The power model was used to assess dose proportionality, and the results are shown in Table 4. The 90% CIs of the slope *β* of C_max_ and AUC_0-t_ were not within the predefined acceptance range, and the direction of the deviation was higher than expected from fitting. The results suggested that the main pharmacokinetic parameter of SAA is the lack of proportionality with the dose range after single dosing.

**TABLE 4:** Assessment of dose proportionality of SAA based on the power model.

	Pharmacokinetic parameter	β	90% CI	Acceptance range	Dose range
Single dose	C_max_	1.214	1.150–1.278	0.918–1.082	20–300 mg
	AUC_0-t_	1.222	1.156–1.288		

SAA, salvianolic acid A; C_max_, maximum plasma concentration; AUC_0-t_, area under the plasma concentration–time curve from 0 to the last measurement time.

### PBPK modeling and human pharmacokinetic prediction

**FIGURE 3:**
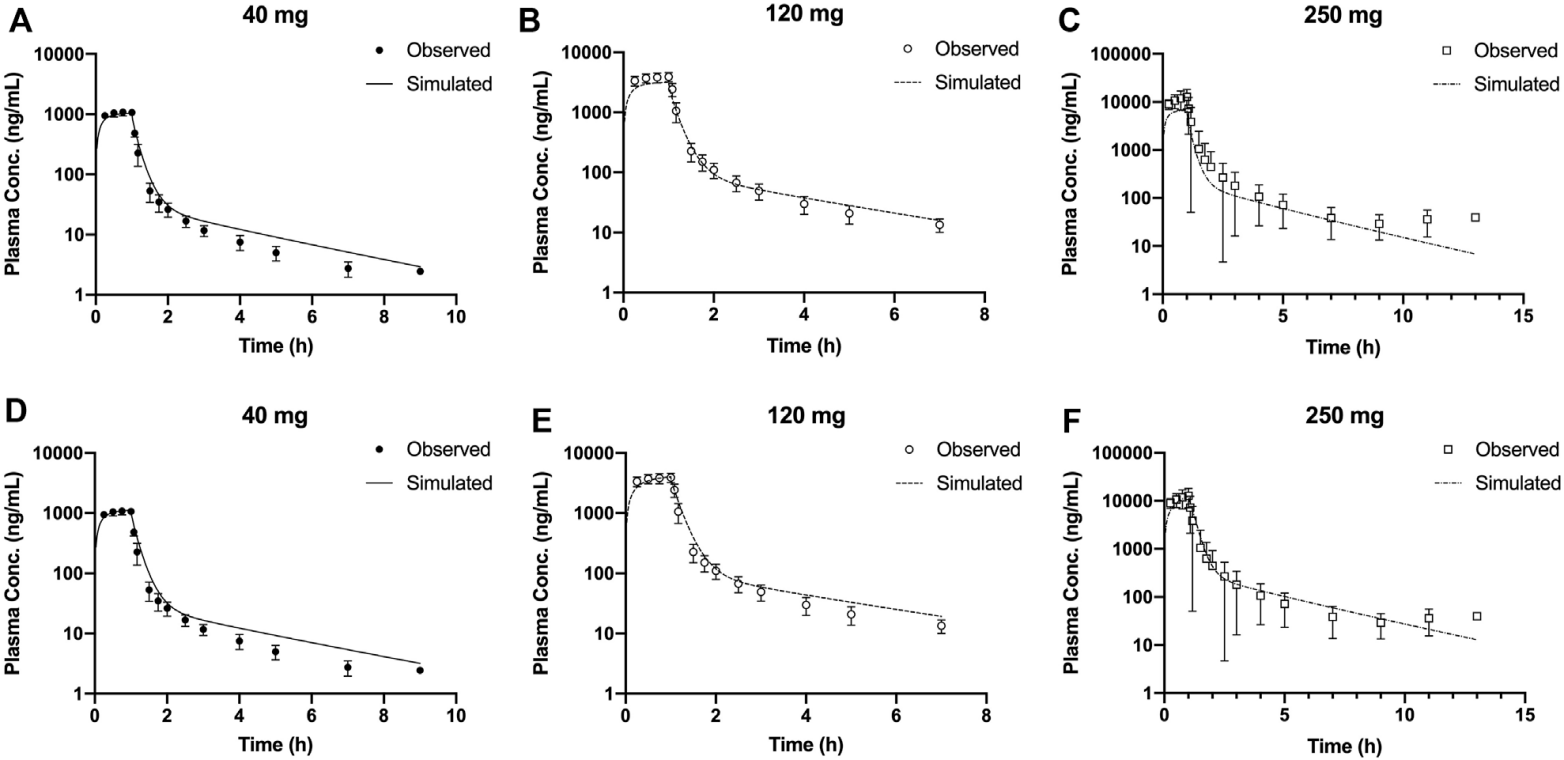
Mean observed (symbol ±SD) and simulated (lines) plasma concentrations for 40, 120, and 250 mg single doses with the original PBPK model **(A–C)** and the updated PBPK model **(D–F)**. PBPK, physiologically based pharmacokinetic.

**FIGURE 4:**
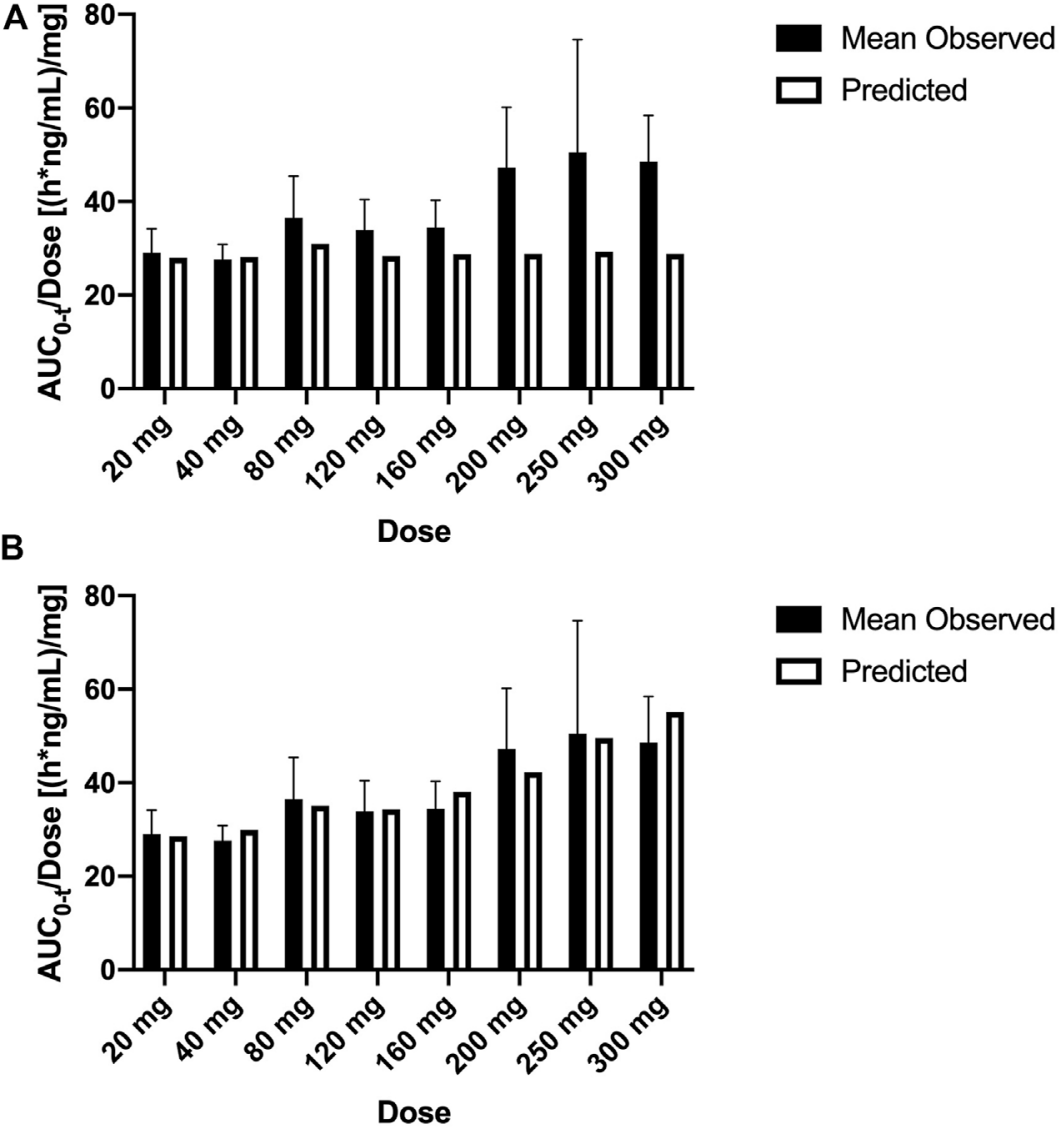
Mean observed (±SD) and predicted dose-normalized AUC_0-t_ for single ascending doses with the original PBPK model **(A)** and updated PBPK model **(B)**. AUC_0-t_, area under the plasma concentration–time curve from 0 to the last measurement time; PBPK, physiologically based pharmacokinetic.

In this study, the PBPK model was optimized and validated in healthy Chinese subjects by using the observed pharmacokinetic data after single dosing. Predicted pharmacokinetic profiles were in good agreement with observed data across the range of doses (20–300 mg); however, the simulation results indicated that the distribution rates of predicted data are faster than those observed at the higher dose level (Figures 3A–C). OATP1B1 and P-gp located in the liver were influx and efflux transporters, respectively, which affect transfer rates of SAA from blood to the liver and from the liver to bile. The transfer ability of these transporters existed at saturation above a certain concentration. The Michaelis–Menten constant (K_m_) of transporters was predicted to be 31.93 μM by ADMET Predictor^®^, and the converted concentration was 15,790 ng/ml. Considering C_max_ was about 14,310 ng/ml at the highest dose, thus, we decreased the K_m_ value and optimized the maximum elimination rate (V_max_). Simulations with the updated model showed a much improved match to plasma concentrations across the whole-dose range **(** Figure 4 **)**. The updated model had only an effect on distribution rate reduction at higher doses, although the K_m_ value was reduced (Figures 3D–F).

**FIGURE 5:**
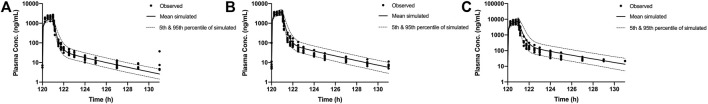
Individual observed day 6 plasma concentrations in healthy Chinese subjects after twice-daily doses (60 mg **(A)**, 120 mg **(B)**, and 200 mg **(C)**, symbols). Solid lines show the mean of PBPK concentrations. The dotted lines represent the 5th and 95th percentiles of simulated concentrations. PBPK, physiologically based pharmacokinetic.

Multiple-dose profiles were measured in 24 healthy subjects to assess three different dose levels of SAA. Day 6 plasma concentrations were measured after twice-daily (Q12H) doses (60, 120, or 200 mg) for 5 days and once on day 6. Two-hundred Monte Carlo simulations were generated using the updated PBPK model and the “population trial” mode in GastroPlus^®^. The results are shown in Figure 5. The observed day 6 AUC_0-t_ of multiple ascending doses (60, 120, and 200 mg) ranged from 1490 to 2620 h*ng/ml, 2858 to 4394 h*ng/ml, and 4731 to 10,318 h*ng/ml, respectively, with mean values of 2126 h*ng/ml, 3601 h*ng/ml, and 6954 h*ng/ml, respectively. For comparison, simulated AUC_0-t_ 5th to 95th percentiles were 1294–2410 h*ng/ml, 2615–5453 h*ng/ml, and 4616–13,510 h*ng/ml, respectively, with mean values of 1705 h*ng/ml, 3682 h*ng/ml, and 7690 h*ng/ml, respectively. The results indicated that multiple ascending dosing was well estimated, and the simulated day 6 concentration profile range almost completely encompassed the observed concentrations.

### Safety and tolerability

In part A, 17 subjects (18.48%) reported 22 TEAEs, including 15 subjects (19.23%) out of 78 subjects and 2 subjects (14.28%) out of 14 subjects who received a single dose of SAA or placebo, respectively. All TEAEs were mild abnormal laboratory test (*n* = 15) and lower blood pressure (n = 2). In part B, 8 subjects (33.33%) out of 24 subjects reported 11 TEAEs. One subject reported moderate phlebitis, which was probably related to intravenous administration. All other TEAEs were mild abnormal laboratory test (*n* = 7), sebaceous cyst (*n* = 1), phlebitis (*n* = 1), and ventricular extrasystole (*n* = 1). The incidence of TEAEs appeared to be not dose-related both in terms of the number of TEAEs and the number of subjects reporting TEAEs.

## Discussion

Cardiovascular and cerebrovascular diseases remain consistently among the leading causes of death worldwide. In China, the use of traditional Chinese medicine in treating such conditions is the most developed field in the modernization of traditional Chinese medicine. Among the many Chinese medicines used for such purposes, Danshen is particularly known for its usage in the treatment of many cardiovascular and cerebrovascular diseases (Ji et al., 2000). SAA and salvianolic acid B (SAB) have been proposed as major contributors to the vasodilative and anticoagulant effects of Danshen extracts; the former has stronger pharmacological activity, acting through the modulation of the calcium influx and the immune response, among other mechanisms (Zhang et al., 2010; Ho and Hong, 2011).

This first-in-human study investigated the safety, tolerability, and pharmacokinetics of single and multiple ascending doses of SAA in healthy Chinese subjects. SAA appeared to be well tolerated with no serious AEs in the study population. The TEAEs observed were mild or moderate in severity, and no dose-limiting AEs were observed during the study.

The pharmacokinetics of SAA was well-characterized in healthy Chinese subjects up to a 300-mg single dose. Different from reports of pharmacokinetic profiles in rats (Sun et al., 2018) and rhesus monkeys (Song et al., 2015), the result of the power model indicated that exposures of SAA in humans lack dose proportionality over the range of 20–300 mg (Table 4). With the increasing SAA dose, exposure increased to a greater extent than the dose increased. Providing a robust criterion for exploratory dose proportionality is difficult, and the power model is still considered to be the suitable approach (Hummel et al., 2009), although the conventional dose proportionality criterion has its limitations due to usually small sample size and large dose range ratio in the phase I study. In this study, PBPK modeling and simulation were applied to investigate the distribution of SAA and try to find a reasonable mechanism for the lack of proportionality of the pharmacokinetic profile with the increasing dose after single dosing.

SAA was classified as Class 3B by ADMET Predictor^®^ based on ECCS. Class 3B compounds are predominantly cleared by hepatic active uptake and/or renal clearance, followed by elimination as an unchanged drug in bile and/or urine (Varma et al., 2012). The OATPs represent a superfamily of important membrane transport proteins that mediate the sodium-independent transport of a diverse range of amphiphilic organic compounds (The International Transporter Consortium et al., 2010). Among these family members, OATP1B1 is predominantly expressed at the basolateral membranes of hepatocytes in the liver and has been implicated to play key roles in the hepatic uptake and plasma clearance of a broad range of drug substrates and toxins (Lee and Ho, 2017). Cao et al. (2019) reported that OATP1B1 plays an important role in SAB uptake in liver cells *in vitro*. As a similar compound, we suggested that OATP1B1 may also affect SAA distribution in humans, and the results of the PBPK model provided some prediction basis. In addition, SAA was mainly methylated by catechol O-methyltransferase (COMT) in the liver and then predominantly eliminated through bile (Xu et al., 2014). We added parameters of OATP1B1 and P-gp, a hepatic efflux transporter to bile, to the PBPK model for simulation. Predicted pharmacokinetic profiles showed much more good agreement with observed data at lower dose levels; however, the simulation results indicated that the distribution rates of predicted data are faster than those observed at the higher dose levels. The result prompted that the transfer ability saturation of the transporter should be considered. Mismatches in the simulated C_max_ and AUC at the higher dose could be improved by reducing the K_m_ value. This change prevented the underestimation of the hepatic transfer rate of SAA from blood at the higher dose. According to the Michaelis–Menten equation, nonlinear characterizations of absorption, distribution, or elimination were shown when the plasma concentrations were larger than K_m_. A reduction in this model parameter has been shown to be more realistic for nonlinear pharmacokinetics. After this model update, simulated intravenous infusion profiles in humans were in close agreement with measured mean plasma levels across the full-dose range (Figure 4), and simulations of multiple-dose pharmacokinetics were also verified as being in good agreement with measured data on day 6 (Figure 5).

PBPK models are based on known pharmacokinetic mechanisms and reflect the current understanding of human physiology and enzymology. This allows major discrepancies of an observed pharmacokinetic profile of a compound from predicted to be interpreted mechanistically. Signature deviations from the observed pharmacokinetic profiles can be useful in the generation of hypotheses and to trigger the right experiments to support the hypotheses (Peters, 2022). In SAA PBPK simulation, the K_m_ value of transporters could affect the simulation accuracy of the pharmacokinetic profile at higher doses. This result suggested that kinetic data on hepatic OATP1B1 and P-gp should be confirmatory studied in liver cells *in vitro*, and the effect of the nonlinear increase in exposure and efficacy at high doses should be considered in the following clinical trials.

## Conclusion

SAA was well tolerated in the dose range studied in healthy Chinese subjects and showed a well-characterized pharmacokinetic profile. The incidence of TEAEs was mild and moderate, which appeared to be not dose-related. Furthermore, the PBPK model could well simulate the observed plasma concentrations and indicated that hepatic OATP1B1 and P-gp may affect dose proportionality. All these findings provided valuable pharmacokinetic knowledge for further clinical development.

## Data Availability

The raw data supporting the conclusion of this article will be made available by the authors, without undue reservation.

## Supplementary material

SUPPLEMENTARY FIGURE S1 Schematic illustration of the blood sampling process. **(A)** Single ascending dose, Part A; **(B)** multiple ascending doses, Part B. “↓”, pre-infusion blood sampling point; “↓”, infusion period blood sampling point; “↓”, post-infusion blood sampling point.

SUPPLEMENTARY FIGURE S2 Mean (± SD) plasma concentration–time profiles, following the single ascending dose of SAA in male (closed circles) and female (open squares) subjects. SAA, salvianolic acid A.

SUPPLEMENTARY FIGURE S3 Mean (± SD) plasma concentration–time profiles, following multiple ascending doses of SAA in healthy Chinese subjects. Each subject received twice-daily doses of SAA for 5 days and once on day 6. SAA, salvianolic acid A.
