# Supplementary figures and images for: First-in-human study to investigate the safety and pharmacokinetics of salvianolic acid A and pharmacokinetic simulation using a physiologically based pharmacokinetic model
Source: Front Pharmacol. 2022 Nov 4;13:907208. doi: 10.3389/fphar.2022.907208 (PMC9672460; doi:10.3389/fphar.2022.907208)

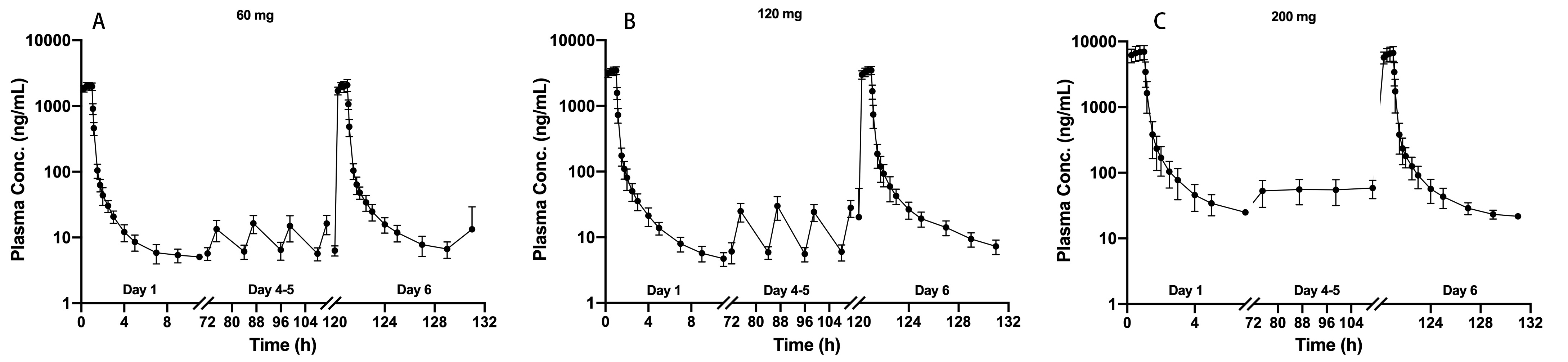

Supplement: Supplementary file 1 [file Image3.JPEG]

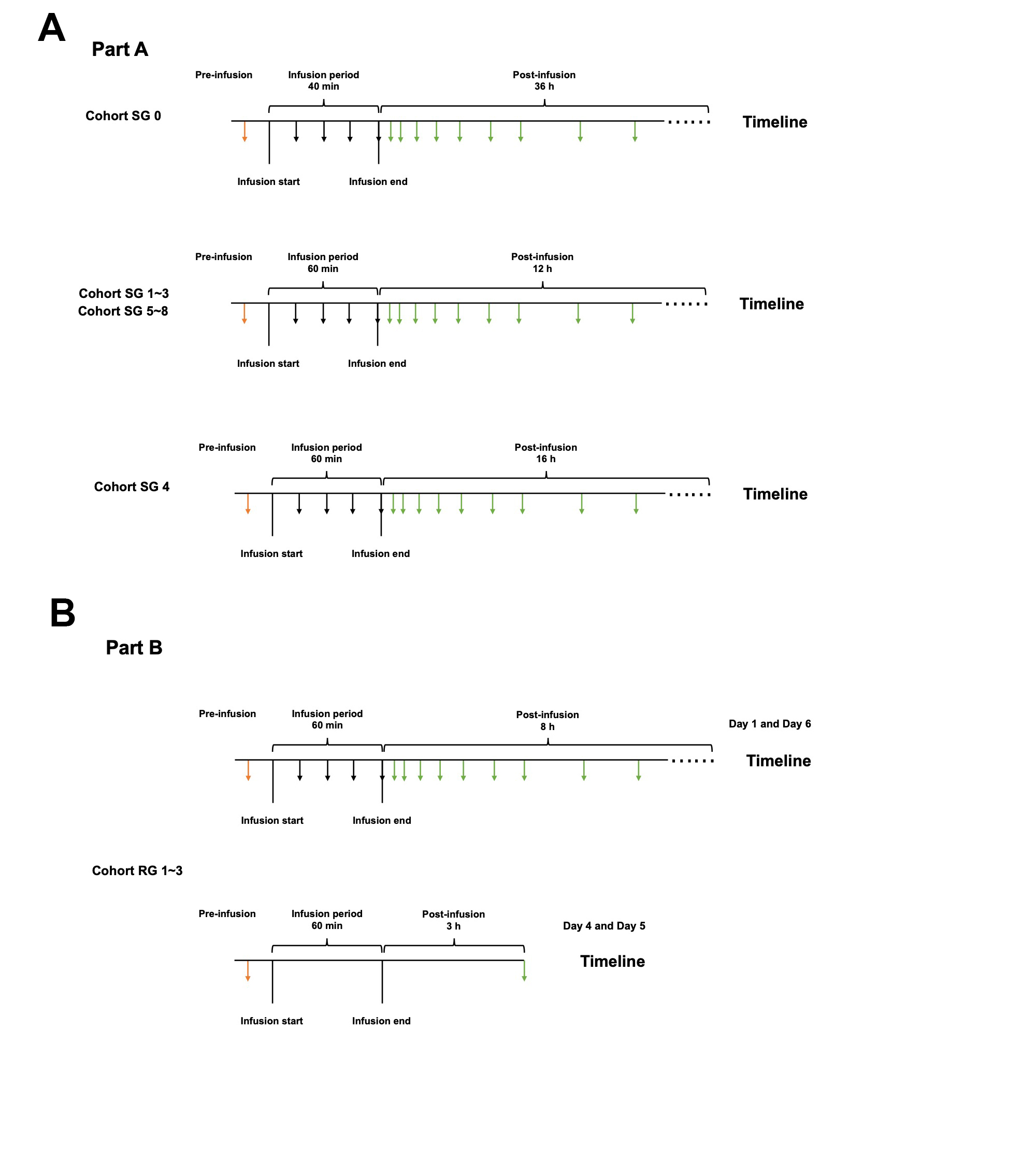

Supplement: Supplementary file 3 [file Image1.JPEG]

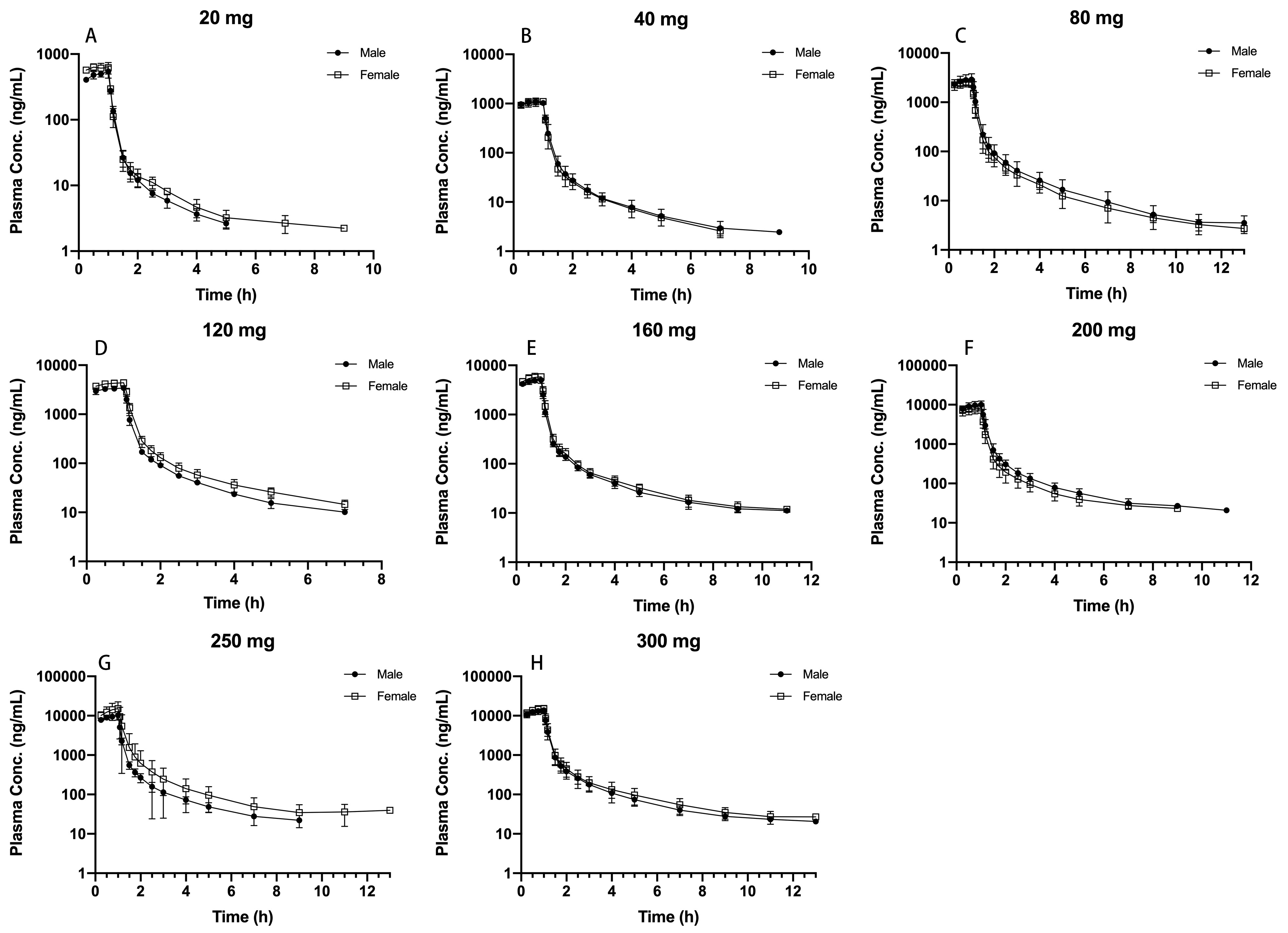

Supplement: Supplementary file 4 [file Image2.JPEG]
