# Supplementary material for: First-in-human study to investigate the safety and pharmacokinetics of salvianolic acid A and pharmacokinetic simulation using a physiologically based pharmacokinetic model
Source: Front Pharmacol. 2022 Nov 4;13:907208. doi: 10.3389/fphar.2022.907208 (PMC9672460; doi:10.3389/fphar.2022.907208)
Supplement: Supplementary file 2 [file Table1.DOCX]

**Table S1.** Physicochemical and in vitro data for SAA

| Parameter | Value | Source |
| --- | --- | --- |
| Molecular Weight (g/mol) | 494.46 |  |
| LogP | 3.89 | Predicted by ADMET Predictor |
| pKa | 3.14 | Measured |
| Solubility (mg/mL) | 0.0849 | Predicted by ADMET Predictor |
| Rbp in human | 0.69 | Predicted by ADMET Predictor |
| Fup in human (%) | 8.57 | Measured |
| OATP1B1, liver |  |  |
| Transporter location | Basolateral/Influx |  |
| In vitro K_m_ (μmol/L) | 2 | Fitted |
| In vitro V_max_ (pmol/min/mg protein) | 50 | Fitted |
| In vitro faction unbound | 1 | Fitted |
| P-gp, liver |  |  |
| Transporter location | Apical/Efflux |  |
| In vitro K_m_ (μmol/L) | 2 | Fitted |
| In vitro V_max_ (pmol/min/mg protein) | 50 | Fitted |
| In vitro faction unbound | 1 | Fitted |
| Pharmacokinetics |  |  |
| SpecPStc (mL/s/mL) | Used, 2×10^-4^ | Fitted |
| Lung: K_p_ | 0.21 | Sun *et al*., 2018 |
| Liver: K_p_ | 0.57 | Sun *et al*., 2018 |
| Spleen: K_p_ | 0.07 | Sun *et al*., 2018 |
| Heart: K_p_ | 0.21 | Sun *et al*., 2018 |
| Brain: K_p_ | 0.27 | Sun et al., 2018 |
| Kidney: K_p_ | 0.28 | Sun *et al*., 2018 |
| PBPK settings |  |  |
| K_p_ Method | Permeability limited tissue,  Rodgers, Leahy, Towland method | Fitted |
| Fu Extracellular/Intracellular Method | Poulin equation | Fitted |
| Tissue albumin | Rothschild | Fitted |

SAA, salvianolic acid A; Rbp, the blood plasma concentration ratio; Fup, fraction unbound; OATP1B1, organic anion-transporting polypeptide 1B1; K_m_, Michaelis-Menten constant; V_max_, the maximum elimination rate; P-gp, P-glycoprotein; SpecPStc, permeability-surface area product per cell volume;

K_p_, tissue-to-plasma partition coefficients; PBPK, physiologically based pharmacokinetic.
